# Primo Vascular System: An Endothelial-to-Mesenchymal Potential Transitional Tissue Involved in Gastric Cancer Metastasis

**DOI:** 10.1155/2015/812354

**Published:** 2015-08-25

**Authors:** An Ping, Su Zhendong, Qu Rongmei, Dai Jingxing, Chen Wei, Zhou Zhongyin, Luo Hesheng, Kwang-Sup Soh

**Affiliations:** ^1^Department of Gastroenterology, Renmin Hospital of Wuhan University, Wuhan 430070, China; ^2^Department of Biochemistry, Nagoya University Graduate School of Medicine, Nagoya 466-8550, Japan; ^3^Department of Anatomy, Southern Medical University, Guangzhou 510515, China; ^4^Nano Primo Research Center, Advanced Institute of Convergence Technology, Seoul National University, Suwon-si 443-270, Republic of Korea

## Abstract

Gastric cancer is the fourth commonest cancer in the world and the second leading cause of cancer-related death. Investigation of gastric cancer metastasis is one of the hottest and major focuses in cancer research. Growing evidence manifested that primo vascular system (PVS) is a new kind of circulatory system beyond vascular and lymphatic system. Previous researches revealed that PVS is a specific tissue between endothelium and mesenchyme and is involved in cancer, especially in tumor metastasis and regeneration. In current study, we investigated the role of primo vessels in gastric cancer metastasis and its possible relationship to vascular vessels formation. Our results indicated that primo vessels were involved in gastric cancer metastasis. We observed blood vessel-mediated metastasis, primo vessel-mediated metastasis, and an intermediate state between them. We deduced that primo vessels may be precursors of blood vessels. These results possibly provided a thoroughly new theoretic development in cancer metastasis.

## 1. Introduction

Gastric cancer is the fourth most common cancer in the world and the second leading cause of cancer-related death despite its declined incidence over the last 50 years [1, 2]. More than 80% of diagnoses occur at the middle to late stage of the disease and metastasis to remote organs frequently is detected [3]. Investigation of gastric cancer metastasis is one of the hottest and major focuses in cancer research. Generally, the metastasis of gastric cancer has three patterns: hematogenous metastasis through vascular vessels, lymphatic metastasis through lymphatic vessels, and implantation metastasis. Recently, growing evidence manifested that primo vascular system (PVS) is a new kind of circulatory system beyond vascular and lymphatic system [4–6]. The functional role of PVS in cancer, especially in tumor metastasis and regeneration, was extensively investigated and recognized by more and more researchers [7–10]. Our previous studies manifested that primo vessels in the mesentery are a specific transitional structure between endothelium and mesenchyme [11]. We deduced that primo vessel cells may be a sort of specific population that had potential capability of transition to endothelial cells or fibroblasts. Therefore, our current study continued to investigate the role of primo vessels in gastric cancer metastasis and its possible relationship to vascular vessels formation.

## 2. Materials and Methods

### 2.1. Cell Line and Cell Culture

The MKN-45 cell line, a poorly differentiated human gastric cancer cell line, was obtained from Korean Cell Line Bank. The cells were maintained in RPMI 1640 (GIBCO, CA) supplemented with 10% fetal bovine serum (GIBCO, CA), 1% penicillin/streptomycin, and 1% L-glutamine (Sigma-Aldrich, St. Louis, MO) and cultured in a humidified incubator in an atmosphere of 5% CO2 at 37°C. For GFP gene transduction, the enhanced GFP4 expression plasmids (Clontech, Palo Alto, CA) were transfected into 90% to 95% confluent MKN-45 cells using Lipofectamine 2000 (Invitrogen, CA), according to the instructions of the manufacturer. Transfected MKN-45 cells (MKN-45-GFP) were cultured into a selective medium that contained 200 mg/mL of G418 (GIBCO, CA). The level of G418 was increased to 1000 mg/mL stepwise. The brightest fluorescent cells (above the 95 percentile) were sorted and cloned by flow cytometry. High GFP-expression populations were isolated with cloning cylinders (Bel-Art Products, Pequannock, NJ) and cultured in the absence of G418 for over 10 passages to select for stable expression of GFP.

### 2.2. Mice

Adult male nude mice (BALB-c-nu/nu, aged 5–7 weeks, weighing 15–20 g, *n* = 20) were obtained from Jung-Ang Laboratory Animal Company (Seoul, Republic of Korea). The animals were maintained in a barrier facility in racks filtered with high-efficiency particulate air filter. All the animal care and studies were approved by the institutional committee on research animal care and were done according to the Korean Policy on Humane Care and Use of Laboratory Animals.

### 2.3. Orthotopic Implantation in Stomach

MKN-45-GFP cells were collected at the log phase and injected subcutaneously into the mice at 10^7^/0.2 mL. Six weeks later, tumors were harvested from the mice under anesthesia (by subcutaneous injection of 0.1 mL of solution of 0.04% Zoletil and 0.06% Rompun) and minced into small pieces (2 × 2 × 2 mm^3^) in HBSS containing 100 U/mL penicillin and 100 *μ*g/mL streptomycin (Figures 2(a)–2(c)). For implantation, the mouse stomach was gently exteriorized via a left-side upper abdominal incision, and one small tissue pocket was formed in the middle wall of the greater curvature using microscissors. One tumor piece was placed into the pocket and closed with a 6-0 surgical suture (Figures 2(d) and 2(e)). Animals were kept in a sterile environment. All procedures of the operation described above were performed with an X7 magnification microscope (Olympus).

### 2.4. Intraoperative Imaging

From 6 to 8 weeks after the orthotopic implantation, abdomens of the tumor-bearing mice were incised. Metastatic tumors and primo vascular system were carefully observed under the X7 magnification microscope. Metastatic tumors and primo vessels with classic appearances of free thread-like and semitransparent structure were captured with a CCD camera (Axiophot, Zeiss, Germany). To detect the GFP fluorescence derived from the gastric cancer, a Leica stereo fluorescence microscope LZ12 (Leica Microsystems, Inc., Bannockburn, IL) equipped with a mercury lamp power supply was used. Under the LZ12 microscope, the GFP fluorescence showed sensitive white color. High resolution images of 1024 × 724 pixels were captured. Images were processed for contrast and brightness and analyzed with the use of Image Pro Plus 4.0 software (Media Cybernetics, Silver Springs, MD).

### 2.5. Phalloidin and DAPI Staining in Tissue Samples

After intraoperative imaging, primo vessels together with metastatic tumors were taken out carefully. To further identify that the primo vessels were involved in gastric cancer metastasis, phalloidin (Invitrogen, CA) and 4′,6-diamidino-2-phenylindole (DAPI, Invitrogen, CA) staining were performed as described previously [11]. Staining was analyzed independently by two investigators using a fluorescence microscope (BX51, Olympus, Japan).

### 2.6. H&E Staining of Metastatic Tumors and Primo Vessels

Subsequently, features of primo vessels and related metastatic tumors were determined by hematoxylin and eosin (H&E) staining. Briefly, metastatic tumors and primo vessels were fixed in 10% neutral buffered formalin (NBF) (pH 7.6) at 4°C. After dehydration and clearing, all the samples were embedded in paraffin and sectioned using a Vibratome Series 1000 sectioning system (Technical Products International, St. Louis, MO) for further H&E staining.

## 3. Results and Discussion

### 3.1. Isolation of Stable, High Level GFP-Expression MKN-45-GFP Cells

**Figure 1:**
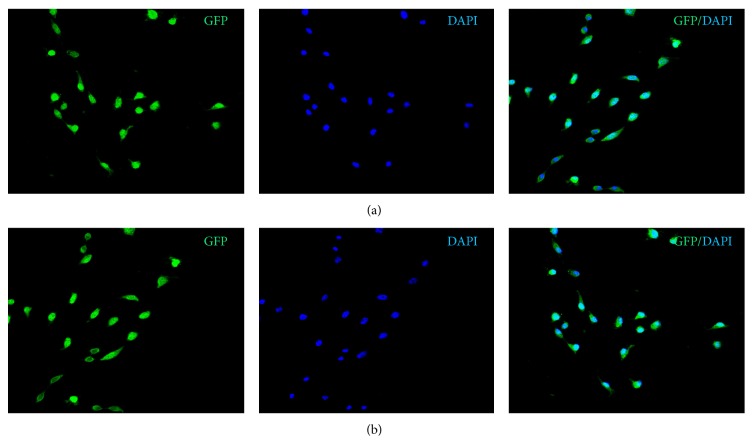
Isolation of stable, high GFP expressed MKN-45-GFP cells. (a) The enhanced GFP4 expression plasmid was transfected MKN-45 cells. The brightest fluorescent cells were sorted and cloned by flow cytometry. Stable high GFP-expression clones were isolated and cultured in the absence of G418 for 10 passages. (b) Stable high GFP-expression MKN-45-GFP cells cultured in the absence of G418 for 20 passages.

In the past, GFP has been used to detect metastatic cells in freshly excised tissue samples [12–14] and for studies of cell motility within primary tumors by in vivo time-lapse confocal microscopy [15]. Studies suggested that the use of GFP-expressing cells could improve in vivo investigations of the metastatic process. Utilization of GFP as a cell label for in vivo experiments requires that cells should be stably transfected and express GFP over a long term. The stable transfected MKN-45-GFP cells used in the present study exhibited a stable and strikingly bright fluorescence (screened by flow cytometry) in the absence of selective agent (G418) after numerous passages (Figure 1). This indicates the suitability and sensitivity of the MKN-45-GFP cells for long term use in vivo.

### 3.2. Stable High GFP-Expression in Gastric Metastatic Tumors

**Figure 2:**
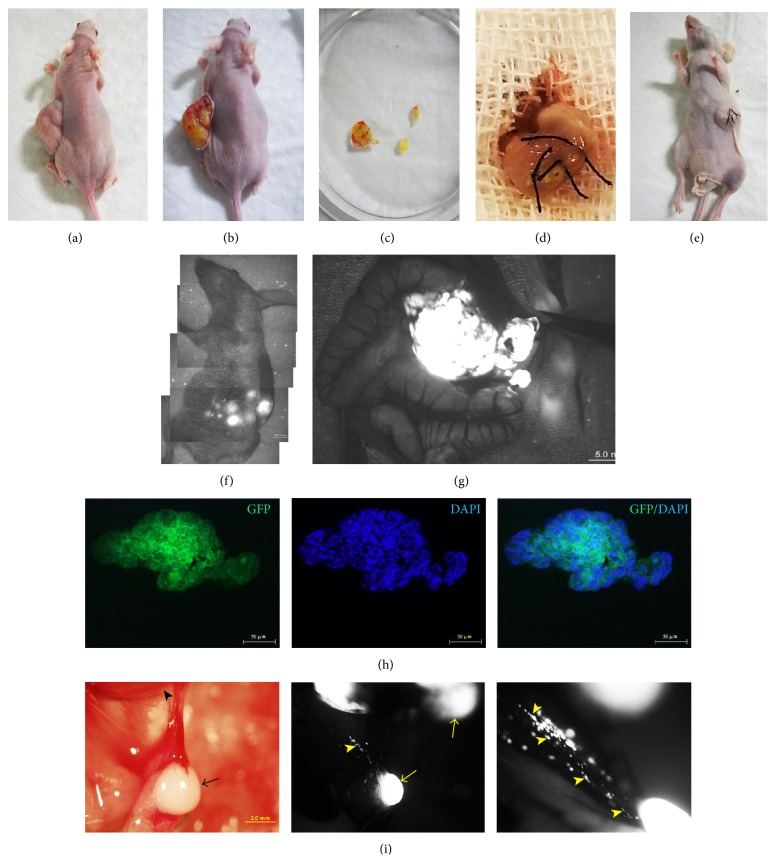
Orthotopic models in vivo and stable high level expression of GFP in gastric cancer metastatic tumors in nude mice. (a) and (b) 5 × 10^6^ MKN-45-GFP cells were inoculated subcutaneously into the left flank of 5–7-week-old anesthetized nude mice. 6 weeks later, subcutaneous tumor grew obviously. (c) Subcutaneous tumor was harvested and incised into small fragments. (d) and (e) Tumor pieces were orthotopically implanted in the gastric wall with surgical suture. (f) and (g) Under the LZ12 microscope, gastric orthotopic tumors expressed strong GFP fluorescence. (h) GFP signals from subcutaneous tumor pieces were tested under fluorescence microscopy. (i) 6 to 8 weeks after the orthotopic implantation, gastric metastasis was investigated. Detection of GFP signal confirmed the metastatic tumors (arrows) and microtumors or gastric cancer cells (arrowheads).

After subcutaneous inoculation of MKN-45-GFP cells in nude mice, tumors were harvested. As shown in Figure 2(h), tumor pieces expressed high GFP fluorescence which further indicated the high quality of stably transfected MKN-45-GFP cells. Then, small tumor pieces were orthotopically implanted into the gastric wall. Six–eight weeks later, metastasis in orthotopic implantation mice was investigated. Under the LZ12 microscope, gastric orthotopic tumors expressed strong GFP fluorescence (Figures 2(f) and 2(g)). Furthermore, images also showed that metastatic tumors, especially multiple micrometastatic tumors, were detected clearly (Figure 2(i)). All these observations suggested successful animal models for gastric cancer orthotopic implantation and metastasis investigations.

### 3.3. Primo Vessels Involved in Gastric Cancer Metastasis

**Figure 3:**
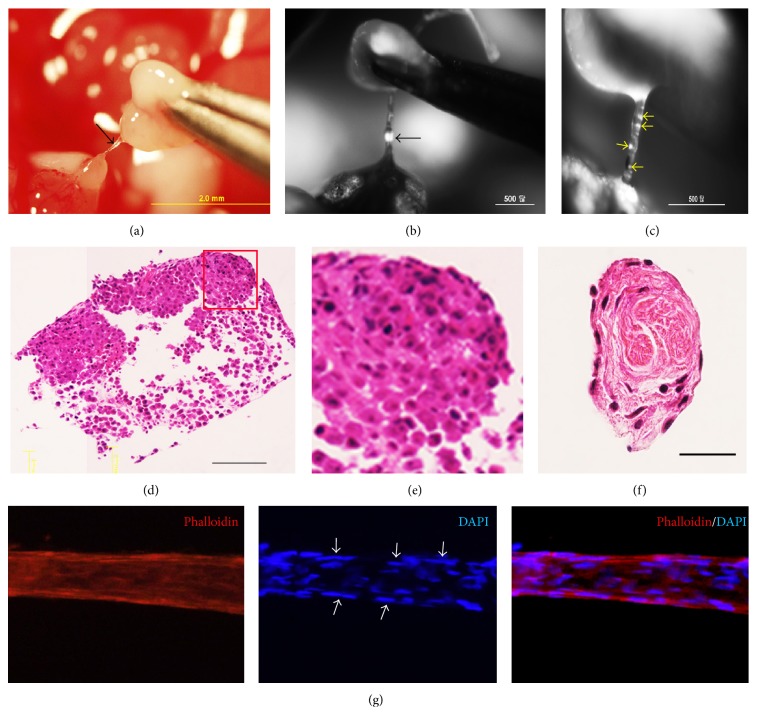
Primo vessels were involved in gastric cancer metastasis. (a) A free primo vessel (black arrow) with a thread-like and semitransparent appearance between tumors. (b) Under the LZ12 microscope, a tiny metastatic tumor (black arrow) was observed in the primo vessel. (c) Magnification of (b); more microtumors or gastric cancer cells (yellow arrows) were detected. (d) HE staining for the metastatic tumor mediated by primo vessels. (e) Magnification of framed area of (d) (Bar: 100 *μ*m). (f) Transversal section of the primo vessel embedded was stained by H&E (Bar: 50 *μ*m). No vascular vessels or lymphatic vessels were observed in either metastatic tumors or primo vessels. (g) Primo vessels involved in gastric cancer metastasis showed rod shape nuclei in a linear arrangement (white arrows) and positively expressed phalloidin.

Next, we investigated the role of primo vascular system in gastric cancer metastasis. We discovered that in orthotopic implantation mice, between the orthotopic tumors and the metastatic tumors, there are free thread-like and semitransparent structures that possess the basic features of primo vessels (Figure 3(a)). Under the LZ12 microscope, besides the fact that metastatic tumors with GFP fluorescence were distinctly detected, tiny tumors in primo vessels were clearly observed (Figure 3(b)). Magnified images revealed multiple microtumors in primo vessels (Figure 3(c)). More importantly, in H&E staining, no vascular vessels or lymphatic vessels were observed in metastatic tumors (Figures 3(d) and 3(e)) and primo vessels (Figure 3(f)). Phalloidin and DAPI staining revealed that these primo vessels expressed a linear arrangement of rod shape nuclei and positive phalloidin expression which conformed with the characters of primo vessels (Figure 3(g)). Accordant with our previous studies, the structure of primo vessels was obviously distinct from that of vascular vessels and lymphatic vessels.

**Figure 4:**
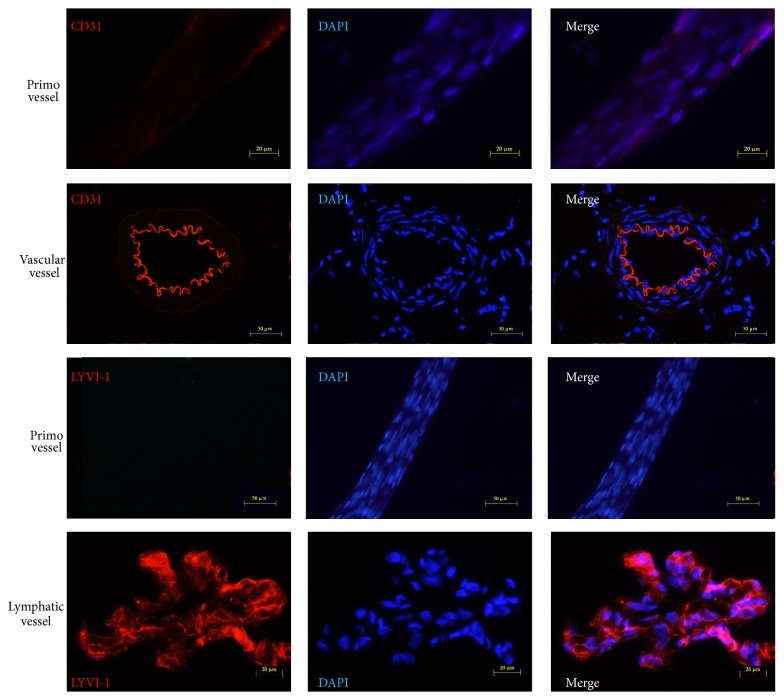
Primo vessels were distinct from vascular vessels and lymphatic vessels. Weak CD31 fluorescence (an endothelium marker) was observed in primo vessels while strong CD31 expression was detected in vascular vessels. Furthermore, primo vessels negatively expressed LYVI-1 (a lymphatic endothelium marker).

We further studied the biologic features of primo vessels involved in gastric metastasis. Our results indicated that contrast to vascular vessels and lymphatic vessels, primo vessels expressed weak CD31 (a endothelium marker) and negative LYVE-1 (a lymphatic endothelium marker) (Figure 4). These data supported our previous opinion that primo vessels belong to a type of endothelial-to-mesenchymal transitional tissues [11] and suggested them as a new metastatic pathway in gastric metastasis. This is another new finding and proof that revealed the role of primo vascular system in cancer metastasis.

**Figure 5:**
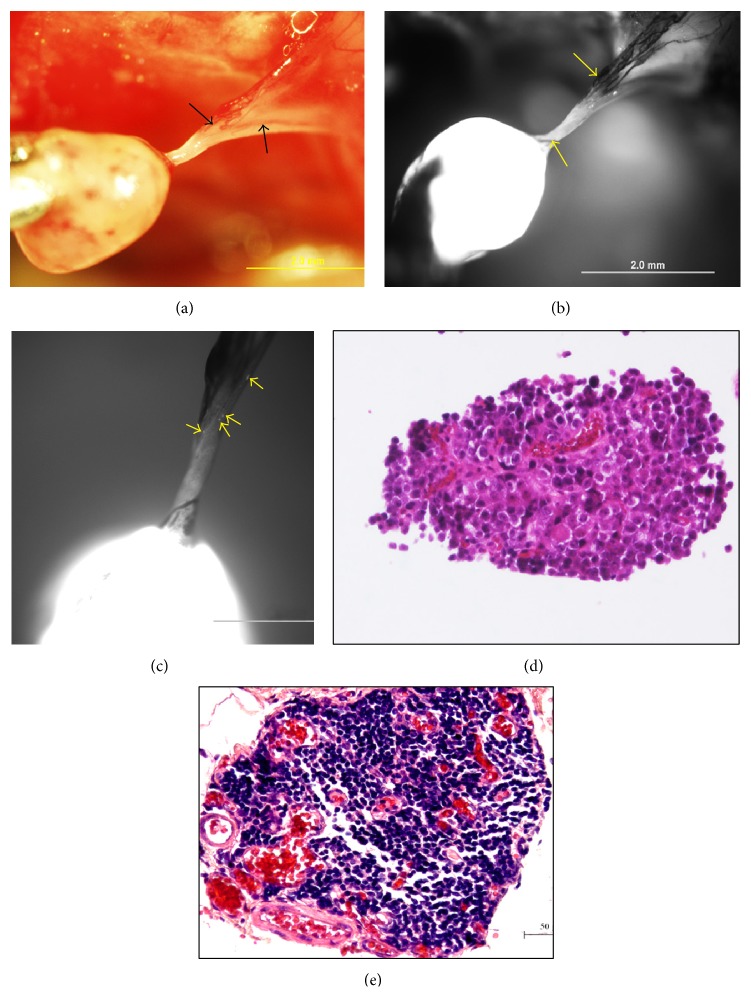
Vascular vessel-mediated metastasis. (a) In vascular vessel-mediated metastasis, metastatic tumors showed orange color and blood vessels were observed (black arrows). (b) Under the LZ12 microscope, metastatic tumor expressed bright GFP fluorescence and vascular vessels were obvious (yellow arrows). (c) Microtumors in the stalk of metastatic tumors were observed. H&E staining revealed blood vessels in the stalk (d) and metastatic tumors (e).

**Figure 6:**
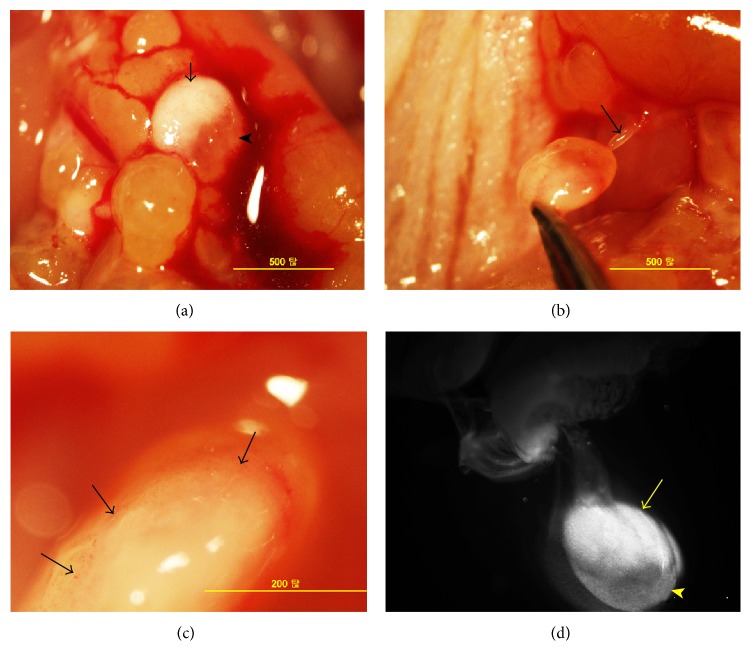
Intermediate state of metastasis between primo vessel-mediated metastasis and vascular vessel-mediated metastasis. (a) Intermediate metastatic tumors between primo vessel-mediated metastasis and blood vessel-mediated metastasis were detected. Half of the tumor showed white color (black arrow) and the other part showed orange color (black arrowhead). (b) Lifting the metastatic tumor in (a); a free, semitransparent, and thread-like structure was detected (black arrow). (c) Magnification of metastatic tumor; red blood vessels and capillaries were obvious (black arrow). (d) Under the LZ12 microscope, high GFP fluorescence was expressed in the proximal part of metastatic tumor (yellow arrow) while lower GFP fluorescence was expressed in the distal part (yellow arrowhead).

Compared with metastasis mediated by vascular vessels, we observed an interesting phenomenon that the brightness of GFP-tagged metastatic tumors was much different between primo vessel-mediated metastasis and vascular vessel-mediated ones. In vascular vessel-mediated metastasis, tumors appeared yellow color (Figure 5(a)) and expressed much brighter GFP fluorescence (Figure 5(b)) while primo vessel-mediated metastatic tumors showed whiter color (Figure 2(a)) but weaker GFP signal (Figure 3(b)). In high magnified images, microtumors in the stalk of metastatic tumors also were observed (Figure 5(c)). H&E staining confirmed vascular vessels in the stalks and metastatic tumors in vascular vessel-mediated metastasis (Figures 5(d) and 5(e)). Interestingly, intermediate metastatic tumors between these two states were detected. As shown in Figure 6, half of the tumor showed white color and the other part showed orange color. Because the cancer cells employed in our experiment were transfected by GFP plasmids, we deduced that the different color indicated different amount of cancer cells in metastasis tumors. Further fluorescence detection revealed the different GFP signals between these two parts (Figure 6(d)).

Our previous study has revealed that primo vessel cells belong to a population between endothelium and mesenchyme [11]. Together with our current observations of the three states in gastric cancer metastasis, we hypothesized that primo vessels may be the precursor for vascular vessels. These results possibly provided a thoroughly new theoretic development in cancer metastasis. Much more work should be done to further reveal the relationship between primo vessels and vascular vessels. We will continue our studies in disclosing the role of primo vascular system in cancer metastasis.

## 4. Conclusions

In conclusion, our research demonstrated that primo vessels are involved in gastric cancer metastasis and may be a potential precursor of vascular vessels. These results possibly provided a thoroughly new theoretic development in cancer metastasis.
